# Concise Review: Stem Cell Models of *SCN1A*-Related Encephalopathies—Current Perspective and Future Therapies

**DOI:** 10.3390/cells11193119

**Published:** 2022-10-04

**Authors:** Valery Zayat, Roza Szlendak, Dorota Hoffman-Zacharska

**Affiliations:** 1 Department of Stem Cell Bioengineering, Mossakowski Medical Research Institute, Polish Academy of Sciences, 02106 Warsaw, Poland; 2 Medical Genetics Department, Institute of Mother and Child, 01211 Warsaw, Poland; 3 Faculty of Biology, Institute of Genetics and Biotechnology, University of Warsaw, 02106 Warsaw, Poland

**Keywords:** *SCN1A*-related disorders, developmental and epileptic encephalopathies, Nav1.1, functional studies, stem cell models, organoids

## Abstract

Mutations in the *SCN1A* gene can cause a variety of phenotypes, ranging from mild forms, such as febrile seizures and generalized epilepsy with febrile seizures plus, to severe, such as Dravet and non-Dravet developmental epileptic encephalopathies. Until now, more than two thousand pathogenic variants of the *SCN1A* gene have been identified and different pathogenic mechanisms (loss vs. gain of function) described, but the precise molecular mechanisms responsible for the deficits exhibited by patients are not fully elucidated. Additionally, the phenotypic variability proves the involvement of other genetic factors in its final expression. This is the reason why animal models and cell line models used to explore the molecular pathology of *SCN1A*-related disorders are only of limited use. The results of studies based on such models cannot be directly translated to affected individuals because they do not address each patient’s unique genetic background. The generation of functional neurons and glia for patient-derived iPSCs, together with the generation of isogenic controls using CRISPR/Cas technology, and finally, the 3D brain organoid models, seem to be a good way to solve this problem. Here, we review *SCN1A*-related encephalopathies, as well as the stem cell models used to explore their molecular basis.

## 1. Introduction

Epilepsy is a chronic disease of the brain, which affects about 50 million people of all ages worldwide, making this disorder one of the most common neurological disorders. It is characterized by the occurrence of repeated and unprovoked seizures, which, in about 30% of cases, could not be adequately treated (WHO; https://www.who.int/news-room/fact-sheets/detail/epilepsy; (accessed on 20 September 2022). Epilepsy is not a single disease but rather a set of disease entities with various etiopathologies, among which those genetically inherited constitute an important group [1]. The monogenic forms of early onset epilepsy currently recognized by the International League Against Epilepsy (ILAE) as developmental and epileptic encephalopathies (DEEs, previously described as early infantile epileptic encephalopathies; EIEEs) are of special research interest because they are characterized by the co-occurrence of epileptiform activity (seizures and EEG abnormalities) accompanied with comorbidities, severe cognitive and behavioral impairments [2,3]. Developmental and/or epileptic encephalopathies, according to the ILAE Task Force on Nosology and Definitions [4], are one of the three categories of epilepsy syndromes beginning in childhood comprising the following syndromes: epilepsy with myoclonic–atonic seizures, Lennox–Gastaut syndrome, developmental and/or epileptic encephalopathy with spike-and-wave activation during sleep, hemiconvulsion–hemiplegia–epilepsy syndrome and febrile infection-related epilepsy syndrome. The etiology-specific classification of epilepsy syndromes signifies DEEs and etiology-specific syndromes in the group of disorders, with onset in neonates and infants (up to 2 years of age), for which there is a specific epilepsy etiology associated with a defined and distinct clinical phenotype in most affected individuals, as well as consistent electroencephalography (EEG), neuroimaging and/or genetic background [5]. Many such disorders are called *channelopathies*, as they are caused by mutations in genes coding ion channels, especially potassium and sodium channels [6]. Here, we would like to concentrate on one of them—Nav1.1, belonging to the voltage-gated sodium channels (VGSCs, Nav) family.

VGSCs have a crucial role in neuronal function controlling the sodium exchange between the extracellular and intracellular spaces. They are essential for the initiation and firing of action potentials. Their principal structural element is a large (∼260 kDa) α subunit, which forms the ion pore and confers the functional and pharmacological properties of the channel, whereas the second element β subunits are involved in modulating the gating and kinetics of the VGSCs [7,8].

Nine different sodium channel α-subunit genes code for Nav1.1–Nav1.9 channel proteins [9]. Four of them, Nav1.1 Nav1.2, Nav1.3 and Nav1.6, encoded by *SCN1A, SCN2A, SCN3A* and *SCN8A* genes, respectively (OMIM; 182389, 182390, 182391, 600702), are involved in the pathogenesis of different forms of epilepsy/developmental and epileptic encephalopathies (OMIM; DEEs Phenotypic Series-PS308350). *SCN1A* had been one of the first recognized “*epilepsy genes*” [10] and is now one of the most frequently identified causes of DEEs [11,12]. This is why *SCN1A* is the most studied gene in terms of the characteristics and pathogenicity of the identified variants. However, the genotype–phenotype correlations for *SCN1A*-related diseases are still not well established. Mutations in the *SCN1A* gene are dominant. According to data of the human pathogenic mutation database HGMD Professional, 2232 mutations were described in the *SCN1A* gene, of which 1984 have the status of pathogenic variants, and 244 are classified as variants of unknown significance (VUS) (HGMD Professional v.2022.1; 06.2022). The vast majority (>80%) of the described pathogenic variants are the basis of *SCN1A*-related DEEs, and they are mainly *de novo* mutations. If variants are inherited, they are derived from parents with somatic and/or germinal mosaicism [13].

The *SCN1A*-related disorders form a broad phenotypic spectrum of epileptic and non-epileptic syndromes. Epilepsy syndromes are characterized by various severities, from relatively mild febrile seizures and generalized epilepsy with febrile seizures plus (FEB3A, generalized epilepsy with febrile seizures plus type 2; GEFSP2/GEFS+, OMIM 604403) to severe forms of DEE, such as the Dravet syndrome (DRVT; OMIM 607208) or developmental and epileptic encephalopathy 6B, non-Dravet (DEE6B; OMIM 619317). The only non-epileptic disorder described so far is familial hemiplegic migraine type 3 (FHM3; OMIM 609634) [14]. Recently, novel *SCN1A*-related phenotypes have been described, including severe DEEs of non-Dravet syndromes: neonatal developmental and epileptic encephalopathy with a movement disorder and arthrogryposis (NDEEMA), developmental and epileptic encephalopathy with a movement disorder (EIDEE/MD), epileptic encephalopathy (EIDEE) [15]. The severe epilepsy DRVT, as well as milder phenotypes associated with GEFS+, are caused by loss of function (LOF) variants. Until recently, gain of function (GOF) variants were associated only with FHM3, but these novel non-Dravet DEEs expand this GOF *SCN1A* diseases spectrum [15,16,17].

**Figure 1:**
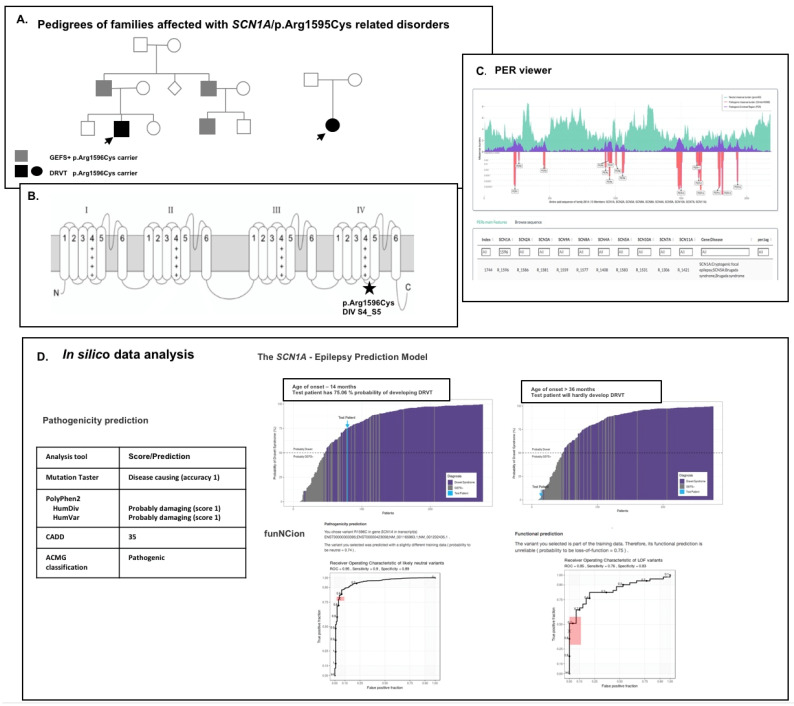
In silico and functional analysis of the missense variant p.Arg1596Cys in the *SCN1A* gene (NM_001202435.1). (**A**) Missense variants in p.Arg1596 are identified in patients of different phenotypes: GEFS+, DRVT and cryptogenic focal epilepsy (HGMD Professional 2022 v.2). The mutation was identified as hereditary in two families, causing GEFS+ and DRVT [24] and sporadic form of DRVT due to *de novo* mutation; (**B**) It is localized in the D4S4–S5 intracellular linker involved in the regulation of fast inactivation of the Nav1.1 channel. The S4–S5 loops coding regions in *SCN1A* are GOF variants enrichment regions [15]; (**C**) The p.Arg1596 residue is outside the PER region of *SCN* paralogs, but substitutions in analogous *SCN5A* residue (p.Arg1583) are responsible for Brugada syndrome (**C**); (**D**) In silico analysis of pathogenicity using different algorithms confirmed the pathogenic character of p.Arg1596Cys variant; however, in this case, the funNCion variant was calculated as probably neutral, and its functional prediction was unreliable. In the *SCN1A* prediction model, there is a strong correlation between prediction and age of onset. In both DRVT and GEFS+ patients, the age of onset was about 14 months, and 36 months and above, respectively. In both cases, prediction recapitulated only the clinical observations. The impact of the p.Arg1596Cys mutation on the functioning of the Nav1.1 channel has previously been shown to result in a complete LOF of the mutated protein [25]. However, in other experiments, this was not so clear cut [26]. These results are difficult to relate with the observed mild and asymptomatic course of *channelopathy* in some carriers and need further validation with the use of other modeling systems.

Encoded by *SCN1A,* the Nav1.1 channel consists of four homologous domains (DI–DIV) composed of six transmembrane subunits (S1–S6). The fourth segment of each domain (S4) plays the role of the voltage sensor and, together with the groove formed by segments S1–S3, in response to membrane depolarization, initiates the transition of the channel to an open state. The segments S5–S6 form the channel pore of ion selectivity fixed by extracellular loops between them. Cytoplasmic loops connecting Nav1.1 DI–DIV domains form a gate involved in the inactivation of open sodium channels (Figure 1). Epilepsy-associated mutations in *SCN1A* are identified along the entire gene/Nav1.1 protein, and there is no correlation between the type of mutation (truncating, missense), its location and a specific phenotype. To evaluate the contribution of different mutations in the *SCN1A* gene to seizure disorders, various in vitro models have been used. Traditionally, these included expression systems [18,19,20] and animal models [21,22,23]. This review will focus on the latest research modeling the pathology of *SCN1A*-related encephalopathies with the iPSC technology.

## 2. Nav1.1/*SCN1A* Mutations Involved in Dravet and Non-Dravet Developmental and Epileptic Encephalopathies

Pathogenic variants in the *SCN1A* gene are among the most common identified genetic causes of most severe childhood epileptic syndromes—DEEs—now recognized as two phenotypically different groups: DRVT and non-Dravet DEEs (NDEEMA, EIDEE/MD, EIDEE). As it turns out, different molecular pathomechanisms are associated with this clinical distinction. In the case of DRVT, we are dealing with LOF mutations, while in the case of NDEEMA, EIDEE/MD and EIDEE syndromes, GOF mutations are changing the channel kinetics. In some individual cases, however, a mixed LOF/GOF effect was also reported. This is the reason why, although classical *SCN1A*-dependent syndromes (DRVT, GEFS+) have been reported for large groups of patients, the consistent prediction of a phenotype–genotype correlation is still difficult, especially for missense variants. As this is not only a scientific dilemma but also an everyday diagnostic problem, in silico prediction tools, such as the *SCN1A*-Epilepsy Prediction Model (https://scn1a-prediction-model.broadinstitute.org/; (accessed on 20 September 2022), funNCion (https://funnc.shinyapps.io/shinyappweb/; (accessed on 20 September 2022) and PER viewer (https://per.broadinstitute.org/; (accessed on 20 September 22022), are being developed to solve it. The first tool calculates the probability of developing DRVT vs. GEFS+ based on a given *SCN1A* variant and the age of seizure onset [15]. funNCion provides a machine-learning method that predicts the effects of genetic missense variants in voltage-gated sodium and calcium channels encoded by *SCNxA* and *CACNA1x* genes for both functional and pathogenicity predictions. The PER viewer is a tool for missense variant interpretation. It allows for identifying pathogenic enriched regions (PERs) across genes and gene families based on analysis of conserved amino acids among gene family members. This algorithm is based on the available data, indicating that the functional characterization of variants in one *SCN* gene may serve as valuable alternative data about the corresponding variants at the analogous positions across different *SCN* genes where subtype-specific functional data are not available [27,28]. However, all these analyses are only theoretical models, not always giving conclusive predictions, and at any rate, they may overestimate disease pathogenicity [29]. An additional problem with the use of in silico tools is the inability to differentiate the phenotypes within a disease spectrum (e.g., GEFS+ vs. DRVT) (Figure 1). This is why the functional/electrophysiological data are more useful in the discrimination of disease severity based on the mutation effect. Variants are categorized either as GOF, LOF or “mixed” functions regarding their biophysical properties; GOF is an increase in Na+ permeability and the persistent current, while LOF is the opposite. The *SCN1A* testing and analysis of Nav1.1 channel functionality are important, as it has been recently shown that it can lead to the implementation of appropriate treatment, e.g., use or exclusion of sodium channel blockers [15].

However, all experimental procedures have their limitations and weaknesses. In the following part of this article, we review what has been learned from *SCN1A* epilepsy models and the possibilities and advantages of the use of patient-specific induced pluripotent stem-cell-derived (iPSC-derived) neurons and 3D organoid models.

## 3. Modeling *SCN1A*-Related Encephalopathies with the iPSC Technology

**Figure 2:**
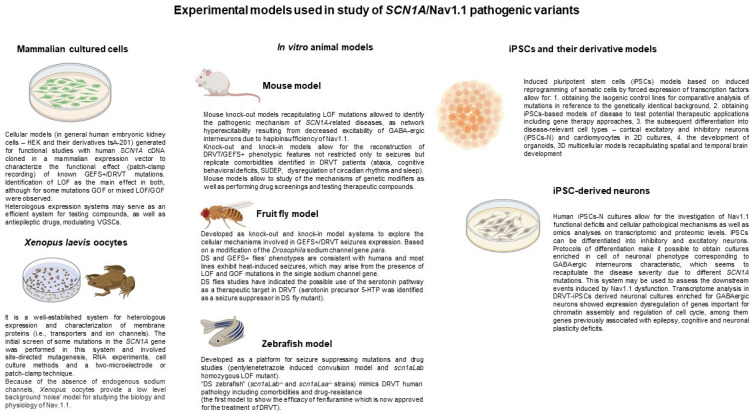
Schematic summary of the experimental models used in research on *SCN1A*/Nav1.1 pathogenic mechanisms based on Refs [31,43,45].

Many model systems for *SCN1A*-related encephalopathies approaches, both in vitro and in vivo, have been successfully used over the years to study genetic epilepsies (Figure 2) [30]. Each model system has advantages as well as disadvantages and is uniquely suited to address a specific set of questions.

The current standard for studying ion channels are mammalian cells transfected with cDNA of the gene and expressing protein under analysis. The ones most frequently used are Chinese hamster ovary cells (CHO) and human embryonic kidney (HEK) cells. Both are of epithelial origin and have small endogenous currents. This is important for patch clamp recordings, but because of the lack of neuronal cell background, the functional studies of ion channels with their application are limited. Additionally, in the case of Na+ channels, they are very sensitive to the cellular background and experimental conditions, which may result in inconclusive results of the mutation’s influence on the channel properties [31]. *Xenopus* oocytes were another widely used ion channel analysis system, which allowed not only heterologous expression but also observation of the functional effects of different compositions of subunits [32]. The main advantage of this system is that these cells do not express a large number of ion channels and receptors, so the exogenous protein can be studied without endogenous contamination. However, the most serious disadvantage, as in other non-neuronal cellular systems, is that the cells are not native cells in which the channels are normally expressed. The Nav1.1 missense variants causative of GEFS+ were studied in the oocyte system, with p.Thr875Met and p.Arg164His showing GOF and LOF changes, respectively [33]. Further analysis of more *SCN1A* variants was also performed for both oocytes and mammalian systems, sometimes showing comparable and sometimes different results [34]. These discrepancies emphasize the necessity to examine the effects of disease-causing mutations in the cell types in which they are normally expressed in vivo. One should also remember the heterogeneity of the clinical picture, even concerning the same mutations, which proves the influence of the genetic background on the expression of the phenotype. This is why the cell line models used to explore the molecular pathology of *SCN1A*-related disorders are only of limited use.

The analysis of the phenotypic differences due to mutations in the *SCN1A* gene and genetic background was possible to carry out in animal models using global knock-out mouse modeling DRVT-related truncated mutations or knock-in missense mutation models. Such mouse models demonstrated that DRVT occurs due to the reduced excitability of GABAergic interneurons, and finally, neuronal network hyperexcitability [35], and those missense mutations identified in GEFS+ and DRVT patients show a common pathogenic mechanism for both disorders. However, the missense mutations cause partial LOF due to Nav1.1 gating modification, and finally, a milder phenotype of the GEFS+ spectrum [31]. Mammalian models, mainly mice, have been widely utilized to study epilepsy mechanisms [30] not only with the use of animals engineered with disease mutations detected in patients. Other animal models were engineered to model chronic epilepsy or acute seizures induced by different stimuli [36].

An analog of the Scn1a+/- knock-out DRVT mouse model is the *scn1Lab* zebrafish (*Danio rerio*) model, harboring a LOF mutation in the sodium channel gene [37]. Phenotypically (behavioral and electrophysiological changes), this model resembles many DRVT characteristics and exhibits a similar response to the ketogenic diet and some anticonvulsant compounds as humans. This is why this zebrafish model was used in phenotype-based drug screening in high-speed imaging studies to unravel the neuronal networks responsible for seizure generation and propagation [38]. The studies revealed that an old antihistamine called clemizole suppressed seizures, and the following analysis indicated that the medication worked by binding to serotonin receptors that mediate neuron excitability.

Another well-known model for human genetic diseases is the fruit fly *Drosophila melanogaster*. In *Drosophila,* there is only one gene encoding VGSC-*para*, producing a variety of sodium channels due to alternative splicing and showing high conservation with human Nav1.1 functional regions. Studies of *para* knock-in and knock-out models show that they exhibit phenotypes that are representative of the *SCN1A*-related disorders (GEFS+, DRVT, depending on the mutation studied), and *Drosophila* may be a model of epilepsy for high-throughput studies of the pathophysiology of diseases and the identification of new biomarkers and treatment options [39,40,41].

Animal models are undoubtedly a valuable tool in research for studying *SCN1A*-related disorders. The results of studies based on such models cannot be directly translated to the affected individuals because they do not address each patient’s unique genetic background. However, this problem can be overcome by using the cell lines derived from individual patients with a specific phenotype, who are carriers of specific mutations in the *SCN1A* gene, e.g., iPSC-derived neurons (iPSC-N). The unique advantage of iPSCs is that they allow for the study of human epilepsy diseases in the context of each person’s “private” genetic makeup, providing a platform for examining the effects of disease-associated genetic variants in the early developmental stages. Patient iPSCs have been used for epilepsy phenotype model diseases associated with *SCN1A*-related encephalopathies [42,43]. Recent advancements in precise gene-editing techniques, including the use of CRISPR/Cas specifically in iPSC lines, have enabled direct evaluation of the association of specific genetic variants with cellular phenotypes [44] (Figure 2).

### 3.1. Modeling the Pathology behind GEFS+ Disease

Several groups have modeled GEFS+ to generate patient-derived iPSC lines to recapitulate the pathology underlying the disease mechanisms. Jiao et al. reported establishing an iPSC line from a patient with p.Gln1923Arg mutation with subsequent differentiation into glutamatergic neurons [46]. The analysis of patient-derived glutamatergic neurons exhibited increased persistent sodium channel activation, as well as increased evoked and spontaneous activity. This was the first evidence to reveal that patient-derived glutamatergic neurons showed a hyperexcitable state and could recapitulate neural pathophysiology, which could be used for screening potential drugs for personalized therapies. Another team reprogramed the fibroblasts of a 10-year-old male affected by partial epilepsy with antecedent FS (PEFS+) caused by the same p.Gln1923Arg heterozygous mutation in the *SCN1A* gene [47]. In the following step, the mutation was corrected using the TALEN-based genome editing (transcription-activator-like effector nucleases) to generate an isogenic control iPSC line to eliminate the potential effect of different genetic backgrounds on the pathology of the disease [48]. The introduction of a fluorescent marker into the first exon of the *GAD67* gene locus, a GABA-synthesizing enzyme, allowed the labeling of the GABAergic neurons in the differentiated neuronal networks and monitoring of the postsynaptic activities of both inhibitory and excitatory neurons [49]. The GABAergic neurons exhibited reduced amplitudes of AP firing and decreased sodium current density, which weakened the spontaneous inhibitory postsynaptic currents in the patient-derived neuronal networks [50]. In a subsequent study, Zhao et al. evaluated the effects of human umbilical cord mesenchymal-stem-cell-conditioned medium (HUMSC-CM) in neurons differentiated from iPSCs derived from the same donor [51]. The treatment with HUMSC-CM reduced cellular deficits, the AP firing was enhanced, and the peak current and the activity of postsynaptic currents were increased. These changes were associated with the improvement of the functional state of the neurons. In addition, this treatment also increased the expression of antioxidant enzyme levels and decreased inflammation and intracellular Ca^2+^ overload. The results suggest that treatment with HUMSC-CM may be a promising therapeutic strategy to improve the clinical deficits in *SCN1A* mutation harboring neurons and warrants further evaluation in clinical studies. Another model was obtained by introducing the same mutation p.Gln1923Arg into the iPSC line from a 16-week-old female fetus by modifying the endogenous *SCN1A* gene using the TALEN-based genome editing tool [52]. As in the previous research, the resulting pair of iPSC lines from the same donor addressed the issue of genetic background noise.

To assess the role of the p.Lys1270Thr *SCN1A* variant in the pathology of GEFS+ disease, Xie et al. generated two pairs of isogenic hiPSC lines by genome editing [53]. The first control pair was generated by reprograming the fibroblasts from an unaffected sibling, subsequently introducing the mutation. The second pair contained the line with the p.Lys1270Thr variant generated from a sample of a GEFS+ sibling with subsequent correction of the mutation. The iPSC lines were differentiated into heterogeneous neuronal populations of both GABAergic and glutamatergic neurons, and their electrophysiological properties were assessed, eliminating the effect of different genetic backgrounds in pairs. The differences between the analyzed pairs of iPSC lines/isogenic controls associated with the genetic background were revealed. The researchers showed that the p.Lys1270Thr substitution caused hyperactive neural networks by altering the sodium current density. It lowered the sodium current density, evoked firing and action potential amplitude in inhibitory GABAergic neurons. On the other hand, it reduced the sodium current density without affecting the evoked firing and AP magnitude in glutamatergic excitatory neurons. Overall, the p.Lys1270Thr variant resulted in hyperactivity in the neural network due to impaired inhibition and excitation in the neurons [53]. To investigate the influence of genetic background on the phenotype, Scalise et al. generated iPSC lines from samples obtained from two siblings coming from a family with the hereditary form of febrile seizures (FS) due to p.Met145Thr substitution in the *SCN1A* gene. That study aimed to obtain a model to investigate the differences in order to explain why, despite the fact that both siblings developed infant FS, only one of them was affected by complex FS, developing temporal lobe epilepsy (TLE) during adolescence [54].

The results of the experiments listed revealed that patient-derived glutamatergic neurons can recapitulate neural pathophysiology (increased persistent sodium channel activation and increased evoked/spontaneous activity) and can be used for screening potential drugs for personalized therapies (Table 1). These results also demonstrate that, using electrophysiological studies, the distinct mutations in the *SCN1A* gene can be correlated with functional deficits evident in the GEFS+ disease.

### 3.2. Modeling Neuronal Deficits Underlying the Dravet Syndrome

As in the case of GEFS+ disease, there are multiple attempts to generate suitable in vitro models to study the DRVT. These include the generation of iPSC lines from patients, correction of the mutations by genome editing, the introduction of mutations into the lines with normal genotypes and manipulation of the *SCN1A* gene.

The first attempt at modeling DRVT with iPSC was carried out in 2013 by Liu and coworkers. The iPSC lines were generated from the fibroblast of two DRVT patients with the *SCN1A* heterozygous splice donor site IVS14+3A > T and nonsense p.Tyr325 * variants. The forebrain-like pyramidal- and bipolar-shaped neurons from those two iPSC lines were generated, showing increased sodium currents, spontaneous bursting and epileptic-like hyperexcitability [55]. Nearly at the same time, other studies reported the generation of DRVT and healthy donor iPSC lines, which were then differentiated into neuronal cells [46,56]. In addition, Chen et al. introduced the p.Gln1923Arg mutation into an iPSC line derived from a donor with a normal genotype [57]. The resulting engineered mutant line was differentiated into an iPSC-N lineage. Additionally, the iPSC-N line from a patient with a p.Phe1415Ile missense variant was generated, and functional characteristics of the glutamatergic neurons were assessed. The patient-derived glutamatergic neurons exhibited increased persistent sodium current and increased spontaneous and evoked activity, but the treatment of neurons with antiepileptic drug phenytoin reduced the functional deficits in persistent sodium currents and action potential firing [46]. Another team generated iPSC lines from DRVT patient fibroblasts with p.Arg1645 * mutation, truncating the fourth homologous domain of Nav1.1 [56]. Upon differentiation into the neuronal lineage, the GABAergic neurons constituted more than half of the cell population, with only a minor fraction of glutamatergic neurons. To select the GABAergic neurons for functional analysis, the researchers created a lentiviral reporter where a fluorescent marker was driven by the *SCN1A* promoter. Electrophysiological studies showed a marked reduction in AP firing in GABAergic neurons with the pathogenic variant compared to the controlled neurons. These functional deficits were even more pronounced with higher current intensities. This was the first stem iPSC-based in vitro model to examine the pathology underlying the DRVT. The same team corrected the p.Arg1645 * mutation by TALEN-based methodology to obtain an isogenic control line [60]. Using a similar strategy, the researchers also generated an iPSC line from a sample of a DRVT donor with a heterozygous nonsense mutation pArg1525 *. This line would provide an additional tool in the study of DRVT, as the functional deficits associated with this mutation have not been reported to date [61]. These results show that glutamatergic neurons derived from both DRVT iPSC lines demonstrated hyperexcitability.

Sun et al. generated iPSC lines carrying the p.Ser1328Pro missense variant obtained from samples of a pair of twins with DRVT [58]. The scientists differentiated those iPSC lines into telencephalic excitatory neurons or medial ganglionic eminence (MGE)-like inhibitory neurons. The inhibitory neurons with the mutation exhibited lower sodium currents, action potential firing and hypersensitivity to inactivation compared to those of control iPSC lines. The functional deficits were rescued by introducing the *SCN1A* transgene, which indicated that a reduction in the Nav1.1 level by RNAi in inhibitory neurons derived from healthy controls could mimic the functional abnormalities associated with DRVT. The excitatory neurons did not show any functional abnormalities. This proof further supported the hypothesis that the deficient inhibitory neurons underlie the pathology of DRVT. Another team generated iPSC lines with and without the p.Val244Leu, p.Lys245 * variants from peripheral blood lymphocytes of an individual with *SCN1A* mutation mosaicism, eliminating the effect of the genetic background behind the pathology of DRVT [42]. The researchers revealed higher levels of tyrosine hydroxylase transcripts and higher concentrations of secreted dopamine in excitatory iPSC-N neurons compared to those of the isogenic control. These findings suggest that a dysfunctional Nav1.1 channel might result in changes in the dopamine system, which contributes to the clinical presentation of DRVT patients. Kim et al. reported two established DRVT iPSC lines with mutations p.Gly1421Trp and p.Ile1194Cysfs*21 [59]. They were used to investigate how distinct *SCN1A* mutations contribute to the functional deficits and the clinical severity of DRVT patients. To address those questions, the iPSC lines were differentiated into forebrain GABAergic neurons, and their electrophysiological properties were studied. Both lines exhibited lower sodium current density and reduced AP firing in GABAergic neurons at higher current intensities. The scientists showed that the sodium current density in GABAergic neurons carrying the missense variant was significantly reduced compared to that of the neurons containing the nonsense mutation. This observation correlated with the severity of the symptoms of both patients. The reason for this phenomenon is not clearly understood but may be due to the fact that the missense mutation might exert a negative-dominant effect during AP firing. This study provides a useful in vitro model to correlate impaired electrophysiological properties of neurons with clinical symptom severity exhibited by the patients. There are some other models of iPSC lines with *SCN1A* DRVT-related mutations under investigation; however, their functional data have not been reported [64,65].

A different research approach was performed by Shuster et al. They generated iPSC lines from the fibroblasts of three DRVT patients with developmental delay and ataxia carrying distinct *SCN1A* mutations: p.Thr217Arg, p.Ala989Pro and p.Pro1837Argfs*24 [43]. The transcriptomes of the derived lines were analyzed during neural differentiation into GABAergic cells at two different time points in comparison to the control iPSC lines generated from healthy subjects. The analysis of the neural progenitor cells and the GABAergic interneuronal cells differentiated from all three DRVT iPSC lines exhibited disturbances in the expression of the genes related to chromatin organization, neural plasticity and excitability pattern compared to the control cells [63]. The GABAergic neurons also showed a change in sodium current activation and an abnormal response to induced oxidative stress compared to control. Deciphering the molecular mechanism underlying the pathology of DRVT in gene expression level along with electrophysiological abnormalities provided the framework for modeling complex neurodevelopmental diseases.

### 3.3. Modeling Cardiac Dysfunctions in the Dravet Syndrome Model

Since most research on modeling pathology behind the *SCN1A*-related encephalopathy focused on identifying the neuronal deficits, it is of particular interest to see to what extent other systems might be involved. This section of the review outlines the current progress in modeling the cardiac deficits associated with DRVT.

Previous research focused on the rate of sudden unexpected death in epilepsy (SUDEP) in DRVT. Cooper et al. reported that out of 100 patients recruited for the study, 10 died of SUDEP [66]. This is probably due to *SCN1A* expression in the heart, not only in the brain, and the dysfunction of Nav1.1 causing cardiac arrhythmia, which contributes to SUDEP in DRVT [67]. To examine this hypothesis and demonstrate how the changes in cardiac excitability may contribute to SUDEP, the researchers differentiated iPSC lines into cardiac myocytes (iPSC-CM). The lines were reprogramed from the samples obtained from four DRVT patients (two with missense p.Arg322Ile; p.Ser1328PPro, two with nonsense p.Arg222 *; p.Tyr325 * variants) and two controls. The four DRVT iPSC lines exhibited increased sodium current and spontaneous contraction rates compared to CM derived from control lines. The most significant increase in the sodium current was observed in the iPSC-CM line derived from the patient who showed cardiac and autonomic deficits upon clinical assessment. To answer the question of whether the mutation in the *SCN1A* gene alone would be sufficient to increase the sodium current, independent of the genetic background, the researchers introduced the p.Phe8Hisfs*91 mutation into the control iPSC line. The results showed that the presence of a heterozygous *SCN1A* truncating variant increased the sodium current [62]. The evidence suggested that the high risk of SUDEP in DRVT patients stemmed from being predisposed to cardiac arrhythmias in addition to GABAergic neuronal hyperexcitability.

## 4. Advantages and Limitations of Stem Cell Modeling Systems

Early onset epilepsy, as opposed to epilepsy later on in life, presents a unique set of challenges associated with developmental and behavioral problems. This also includes antiseizure medication, where therapeutic choices for intervention are less available for infants and neonates. The advances in deciphering the molecular basis of many DEEs, such as DRVT, will bring forward targeted therapies. What complicates the picture is often unremarkable clinical presentation in the younger population, especially neonates. The advent of stem cell technologies allows us to gain an understanding of specific pathologies associated with early onset neurodevelopmental disabilities. Now, establishing iPSC-N lines and human cortical organoid models give us the best chance to recapitulate the early embryonic events and evaluate what underlies the pathology in forming neural circuitry and synaptogenesis. The cortical organoid model in conjunction with cutting-edge technologies will allow us to understand the molecular underpinning of the pathology and possible ways of therapeutic interventions. Furthermore, another advantage of the reprograming of somatic cells to produce iPSC is that they require only a tissue biopsy for derivation, so we can avoid the ethical problems specific to the derivation of pluripotent stem cell lines from oocytes and embryos [68].

It should also be mentioned that the iPSC line generating technology comes with its own set of challenges. Large deletions and insertions associated with genome editing technology have been uncovered. In addition, aneuploidy and chromosomal truncations are also often seen with Cas9-mediated cleavage [69]. Simkin et al. attempted to address the issue of establishing a quality control workflow for the generated iPSC lines. The scientists examined 27 iPSC clones generated from patients with various mutations, including the *SCN1A* nonsense variant p.Gly891Efs*3. It was uncovered that in about one-third of the cases, the iPSC lines acquired large genomic aberrations along with insertions and loss of heterozygosity. What is important is that all these defects escaped the mainstream sequencing analyses. The researchers outlined a cost-efficient quality control strategy, which would minimize the occurrence of such chromosomal aberrations [45].

## 5. Conclusions

*SCN1A*-related disorders encompass not only GEFS+ and DEEs, such as DRVT, caused by LOF mutations, but also newly characterized neonatal syndromes (non-Dravet, DEE6B), expanding the GOF mutation spectrum. This review emphasizes the importance of identifying the distinct mutations present in the *SCN1A* locus and their relevance to the functional deficits displayed by the patients, which is especially important in cases of missense variants identified in the early stages of disease development and in making therapeutic decisions about treating a patient more or less aggressively.

It is also worth noting that now, the European Bank of induced pluripotent Stem Cells (EBiSC) offers eight human iPSC lines with distinct mutations in the *SCN1A* gene for research purposes. Among those are five missense (p.Thr105Ile, p.Ala371Val, p.Thr1721Lys, p.Arg1245 *, p.Cys1741Ser) and three frameshift (p.Ser550Phefs*20, p.Val1352Cysfs*5, p.Tyr1508fs*4) mutations. The Human Pluripotent Stem Cell Registry offers the iPSC line with *SCN1A* p.Arg865 * and p.Ile110Valfs*10 mutations. The functional deficits of the available iPSC lines are not yet reported. The availability of such lines will give more researchers access to the system models to access the functional ramifications of distinct *SCN1A* mutations.

## Figures and Tables

**Table 1:** The timeline represents the historic order of the iPSC-based models in the study of *SCN1A*/Nav1.1 pathogenic variants.

Year	Pathogenic Variant	Origin of iPSC Line	Diagnosis	Advantages and Limitations	Ref.
2013	p.Gln1923Arg p.Phe1415Ile	Patient Patient	GEFS+ DRVT	Differentiation into glutamatergic neurons; hyperexcitable state of enlarged and persistent sodium channel activation in both lines	[46]
2013	IVS14+3A>T p.Tyr325 *	Patient Patient	DRVT DRVT	Differentiation into forebrain-like pyramidal- and bipolar-shaped neurons; hyperexcitability in both types of neurons in both lines	[55]
2013	p.Arg1645 *	Patient	DRVT	Differentiation primarily into GABAergic neurons; impairment in action potential generation	[56]
2014	p.Gln1923Arg	Healthy donor	DRVT	Introduction of mutation into a healthy control line; differentiation into an iPSC-N lineage with normal functional characteristics	[57]
2016	p.Ser1328Pro	Patient	DRVT	Differentiation into telencephalic excitatory neurons or medial ganglionic eminence (MGE)-like inhibitory neurons; functional deficits in inhibitory neurons	[58]
2016	p.Val244Leu; p.Lys245 *	Patient + isogenic control	DRVT	Changes in dopamine system and higher tyrosine hydroxylase mRNA/protein in excitatory iPSC-N	[42]
2018	p.Gly1421Trp p.Ile1194Cysfs*21	Patient Patient	DRVT DRVT	Functional impairment in GABAergic neurons in both lines	[59]
2018	p.Arg1645 *	Isogenic control	DRVT	Normal functional characteristics	[60]
2018	pArg1525 *	Patient	DRVT	Lack of functional studies	[61]
2018	p.Arg322Ile p.Ser1328PPro p.Arg222 * p.Tyr325 * p.Phe8Hisfs*91	Patient Patient Patient Patient Healthy donor	DRVT DRVT DRVT DRVT DRVT	Differentiation into cardiac myocytes (iPSC-CMs); deficits in functional studies in all four DRVT iPSC-CMs (exhibited increased sodium current and spontaneous contraction rates) Introduction of mutation into a healthy control; functional deficits (increased sodium current)	[62]
2019	p.Thr217Arg p.Ala989Pro p.Pro1837Argfs*24	Patient Patient Patient	DRVT + developmental delay and ataxia	Differentiation into neural progenitor cells and the GABAergic interneuronal cells; functional deficits in GABAergic interneuronal cells generated from all three iPSC lines; disturbances in the expression of the genes related to chromatin organization, neural plasticity and excitability pattern	[43,63]
2020	p.Tyr1102 *	Patient	DRVT	Lack of functional studies	[64]
2020–2022	p.Gln1923Arg	Patient	GEFS+	Generation of isogenic control; labeling of the GABAergic neurons in the differentiated neuronal networks and monitoring of the postsynaptic activities of both inhibitory and excitatory neurons; functional deficits in GABAergic neurons; HUMSC-CM reduced cellular deficits and enhanced the AP firing	[47,48,49,50,51]
2020	p.Gln1923Arg	Healthy donor	Normal genotype	Introduction of mutation into a healthy control; lack of functional studies	[52]
2020	p.Lys1270Thr	Patient + isogenic control	GEFS+	Generation of two pairs of isogenic iPSC lines from mutated iPSC and healthy control, respectively; differentiation into heterogeneous neuronal populations of both GABAergic and glutamatergic neurons; impaired inhibition and excitation in neurons	[53]
2020	p.Met145Thr	Patient	FS/TLE	Lack of functional studies	[54]
2021	*SCN1A* knockout	Healthy donor	Normal genotype	Lack of functional studies	[65]
2022	p.Gly891Glufs*3	Patient	DRVT	In one-third of 27 iPSC lines, including the p.Gly891Efs*3 line, large genomic aberrations and loss of heterozygosity were detected	[45]
	p.Thr105Ile p.Ala371Val p.Thr1721Lys p.Arg1245 * p.Cys1741Ser p.Ser550Phefs*20 p.Val1352Cysfs*5 p.Tyr1508fs*4	Patient Patient Patient Patient Patient Patient Patient Patient	DRVT DRVT DRVT DRVT DRVT DRVT DRVT DRVT	European Bank of induced pluripotent Stem Cells	EBiSC
	p.Arg865 * p.Ile110Valfs*10	Patient Patient	DRVT DRVT	The Human Pluripotent Stem Cell Registry	hPSCreg

## Data Availability

Not applicable.
